# Direct Transformation of Lung Microenvironment by Interferon-α Treatment Counteracts Growth of Lung Metastasis of Hepatocellular Carcinoma

**DOI:** 10.1371/journal.pone.0058913

**Published:** 2013-03-18

**Authors:** Peng-Yuan Zhuang, Jun Shen, Xiao-Dong Zhu, Ju-Bo Zhang, Zhao-You Tang, Lun-Xiu Qin, Hui-Chuan Sun

**Affiliations:** 1 Department of General Surgery, Xinhua Hospital, School of Medicine, Shanghai Jiao Tong University, Shanghai, People’s Republic of China; 2 Liver Cancer Institute and Zhongshan Hospital, Fudan University, Shanghai, People’s Republic of China; University of Hong Kong, Hong Kong

## Abstract

**Background:**

Interferon (IFN)-α is effective in inhibiting tumor growth and metastasis of hepatocellular carcinoma (HCC). However, the biologic mechanisms of IFN-α treatment in lung metastasis are not yet clear.

**Methods:**

The effect of IFN-α treatment was studied by using an orthotopic xenograft model and measuring tumor size and lung metastasis. Pretreatment with IFN-α before implantation of tumor was done to explore the effect of IFN-α on lung tissues. Cytokines and macrophages were measured by immunohistochemistry and/or PCR assay, using human origin or mouse origin primers to differentiate the sources. Circulating tumor cells (CTCs) were also assayed by flow cytometry.

**Results:**

IFN-α treatment did not decrease the number of CTCs (0.075%±0.020% versus 0.063%±0.018%, *P* = 0.574, IFN-α–treated versus control groups), but did decrease the number and size of lung metastasis (number: 1.75±1.0 versus 28.0±6.3, *P* = 0.008; size [pixels]: 116.8±72.2 versus 5226.4±1355.7, *P* = 0.020), and inhibited macrophage infiltration (0.20%±0.04% versus 1.36%±0.21%, *P* = 0.0058) and alteration of matrix metalloproteinase (MMP)-9 expression (mean integrated optical density (IOD): 5.1±1.7 versus 21.9±0.4, *P*<0.000) in the lung, which was independent of the primary tumor.

**Conclusion:**

IFN-α inhibited lung metastasis by directly modulating the lung microenvironment.

## Introduction

Hepatocellular carcinoma (HCC) is the sixth most common cancer and the third most common cause of death from cancer worldwide [1], [2]. Patient survival has been improved with recent advances in diagnostic and therapeutic modalities in patients with resectable HCC [3]. However, many patients with advanced or metastatic HCC are not candidates for surgery, and systemic chemotherapy is far from satisfactory [4], [5]. The lung is one of frequent sites of extrahepatic recurrence after hepatectomy, which remains the major obstacle for further improving the survival of patients with HCC after surgical treatment [2], [6], [7], [8].

Interferon (IFN)-α has a variety of biologic properties, including antiviral, immunomodulatory, anti-proliferation, and anti-angiogenic effects [9], [10]. Previous studies showed that IFN-α exerts an inhibitory effect on HCC growth mainly through anti-angiogenesis by down-regulation of vascular endothelial growth factor (VEGF)-A[10], [11], [12], [13], [14]. Recent studies reported the survival benefits of IFN-α monotherapy and IFN-α–based combination therapy for advanced HCC with extrahepatic lung metastasis or tumor thrombi in the major trunk [15], [16], [17]. Adjuvant IFN-α treatment is effective in patients with HCC after hepatectomy or ablation, primarily by postponing or decreasing tumor recurrence and lung metastasis [18], [19], [20], [21], [22], [23], [24]. In a clinical study, we noticed that withdrawal of IFN-α treatment usually resulted in a higher rate of tumor recurrence or lung metastasis as compared with continuous IFN-α treatment [24]. These results suggested that tumor cells, as the seeds of recurrence and metastasis, may survive IFN-α treatment by acquiring additional molecular and biologic changes in response to the pressure of IFN-α treatment.

The interaction between tumor cells and the lung microenvironment may be the key factor in determining the fate of lung metastasis [25]. Recent studies have recognized that macrophages and matrix metalloproteinase (MMP)-9 expression play a critical role in the growth of metastatic lesions in lung tissue [26], [27], [28]. However, the impact of IFN-α treatment on the interaction between metastatic tumor cells and the lung microenvironment has not been reported.

In the present study, we used an orthotopic xenograft model [29], [30], [31]and found that IFN-α treatment could directly modulate the lung microenvironment by reducing macrophage infiltration and MMP-9 expression, which made the lung resistant to the disseminated HCC cells and inhibited metastatic growth.

## Materials and Methods

### Animals, Cell Culture, and Drugs

Six-week-old male BALB/c nu/nu nude mice weighing approximately 20 g (Shanghai Institute of Materia Medica, Chinese Academy of Sciences, Shanghai, PR China) were housed in laminar flow cabinets under specific pathogen-free conditions. The mice were cared for in accordance with the National Institutes of Health Guidelines for the Care and Use of Laboratory Animals. The experimental protocol was approved by the Shanghai Medical Experimental Animal Care Committee (Approval Number: 2009–3556).

HCCLM3 human HCC cells [30], [31] were grown as a monolayer culture in Dulbecco’s modified Eagle’s medium supplemented with 10% bovine serum albumin. Stable red fluorescent protein (RFP)-expressing HCCLM3 cells[29] were maintained in the same culture as the HCCLM3 cells. All cells were cultured at 37°C in a 5% CO_2_, 95% air environment in humidified incubators.

### Orthotopic Nude Mouse Model

The nude mouse model was established by orthotopic implantation of a histologically intact tumor tissue derived from HCCLM3 or RFP-LM3 cell lines, which had been maintained for more than 80 passages in nude mice. All HCCLM3 and RFP-LM3 models exhibited 100% transplantable characteristics and lung metastatic ability as well as various manifestations reminiscent of tumor behavior in patients with HCC.

In the first experiment, when the average tumor volume had reached 100 mm^3^ approximately 1 week after implantation, IFN-α (Sinogen, Kexing Bioproduct Company Ltd. Shenzhen, PR China, at a dose of 1.5×10^7^ U/kg per injection) or vehicle (normal saline, NS) was injected subcutaneously daily in two groups of mice (*n* = 6) for 6 weeks. IFN-α withdrawal study was conducted with daily IFN-α treatment for 3 weeks in one group (six mice) followed by daily NS injection for 3 weeks. All mice were sacrificed after 6 weeks of treatment. Tumor size was assessed using the formula: width × length × depth × π/6. Body weights were measured weekly as a surrogate for toxicity.

In another animal study using nude mice without tumors, each group of mice (*n* = 5 in each group) received daily subcutaneous IFN-α or NS injection for 6 weeks, or IFN-α injection for 3 weeks followed by NS injection for another 3 weeks. After 6 weeks of treatment, all mice were sacrificed.

### Experimental Lung Metastatic Model

In an experimental metastasis study, two groups of mice (*n* = 5 in each group) were pretreated with IFN-α or NS for 3 weeks; then all mice were injected with 1×10^6^ RFP-LM3 cells via the tail vein. Afterward, both groups received 6 weeks of NS treatment, after which the lungs were removed for further study.

### Assessment of Lung Tissue

Fresh lung tissue was transversely cut into two halves; one half was preserved in 10% buffered formalin for immunohistochemistry staining and the other was used in real-time PCR studies. The fresh lung tissue was examined under fluorescence microscopy for lung metastasis, and then representative lung metastatic tissue was confirmed by hematoxylin and eosin (H&E) staining on tissue sections[29]. Results for lung metastasis were consistent between RFP detection and H&E staining.

### Immunohistochemical Studies

Deparaffinization and rehydration of tumor and lung sections were followed by treatment of the sections with 0.3% H_2_O_2_. Sections were incubated overnight at 4°C with primary antibody; after the primary antibody was washed off, the components of the Envision-plus detection system were applied with a polymer (EnVision+/HRP/Mo, Dako, Glostrup, Denmark). Reaction products were visualized by incubation with 3,3′-diaminobenzidine. The following anti-mouse primary antibodies were used: anti-MMP-9 (Abcam, Cambridge, MA), anti-F4/80 (Serotec, Raleigh, North Carolina, USA), anti-interleukin (IL)-10 (Abcam), and anti-IL-12 (Abcam), CD86(Abcam), CD163(Abcam). The scoring of immunohistochemistry staining was conducted as previously prescribed [32]. Briefly, under high-power view, images of four representative fields were captured by the Leica QWin Plus v3 software (Leica Microsystems Imaging Solutions, Cambridge, UK) using identical image system settings, and integrated optical density (IOD) (pixels) was measured by Image-Pro Plus v6.2 software (Media Cybernetics, Bethesda, MD). Macrophage density was formulated as the positive area divided by the total area of each picture.

Primary antibodies for immunofluorescent staining were a mouse monoclonal anti-F4/80 antibody (1∶100, Zymed Laboratories, San Francisco, CA), a rabbit monoclonal anti-MMP-9 antibody (1∶250, Abcam), a mouse monoclonal anti-inducible NO synthase (iNOS) antibody (1∶200, Abcam), a rabbit monoclonal anti-Arginse-1 (Arg-1) antibody (1∶200, Santa Cruz Biotechnology, Santa Cruz, CA). Primary antibodies were detected by using secondary antibodies of anti-mouse IgG- Texas Red (TR) (Santa Cruz Biotechnology) and anti-rabbit IgG-fluorescein isothiocyanate (FITC) (Santa Cruz Biotechnology), respectively. Frozen lung sections (8 µm) were air-dried, hydrated with phosphate-buffered saline (PBS), blocked with 10% goat serum in PBS for 30 min, and incubated with primary antibodies overnight at 4°C. Sections were washed three times in PBS, followed by secondary antibody for 1 h at room temperature. After washing in PBS, sections were mounted with anti-fade reagent with 4′,6-diamidino-2-phenylindole (DAPI) (Invitrogen,USA) and viewed with a fluorescent microscope (×20 objective magnification, Olympus).

### Real-Time PCR

To detect the mRNA level of VEGF-A, platelet-derived growth factor (PDGF)-A, IL-6, proliferating cell nuclear antigen (PCNA), MMP-9, IL-10, IL-12, IFN-α, iNOS and Arg-1 in the lung tissue, the total RNA was extracted following the manufacturer’s protocol (Invitrogen), and 1 µg of total RNA was reverse transcribed using the Primescript^TM^ RT reagent kit (TaKaRa, Otsu, Shiga, Japan). Real-time PCR analysis for quantification was performed using a SYBR Premix Ex Taq^TM^ (perfect real time) (TaKaRa). The relative mRNA expression was normalized to GAPDH. The reactions were run in triplicate using the following conditions: one cycle at 95°C for 10 s, subsequently, 40 cycles were performed at 95°C for 5 s and 60°C for 30 s. The median Ct value was determined, and data were expressed as fold change of relative mRNA expression using the comparative Ct method. Primers used are listed in Table S1.

### Fluorescence-Activated Cell Sorting (FACS) Analysis

The number of circulating tumor cells (CTCs) was determined by flow cytometry. Briefly, 100 µL of blood was collected via heart puncture when each mouse was sacrificed. Red cells were lysed with 500 µL of lysing buffer (Becton Dickinson, Mountain View, CA), vortexed, and resuspended in 750 µL of PBS. Samples were then subjected to a two-dimensional side scatter-fluorescence histogram analysis with a FACS instrument (Becton Dickinson). Blood from mice bearing tumors without RFP expression was used as the negative control. The quantity of CTCs was expressed as a percentage of the total cells.

### Statistical Analysis

Analysis was performed with SPSS 13.0 for Windows; the Pearson χ^2^ test or Fisher exact test was used to compare qualitative variables; and quantitative variables were analyzed by the Student *t* test. Spearman correlation coefficient (cc) determination was used to analyze the correlation among the marker expressions. Kaplan-Meier analysis was used to determine the overall survival. The log-rank test was used to compare survival outcomes between subgroups, and *P*<0.05 was considered statistically significant.

## Results

### IFN-α Inhibited Tumor Growth and Lung Metastasis

IFN-α treatment significantly inhibited tumor growth. After 6 weeks of treatment, tumor volume was 11,959.0±1715.4 mm^3^ versus 1730.2±369.3 mm^3^ (*P*<0.000) for control and IFN-α–treated groups respectively, but IFN-α did not induce any significant loss of body weight.

**Figure 1:**
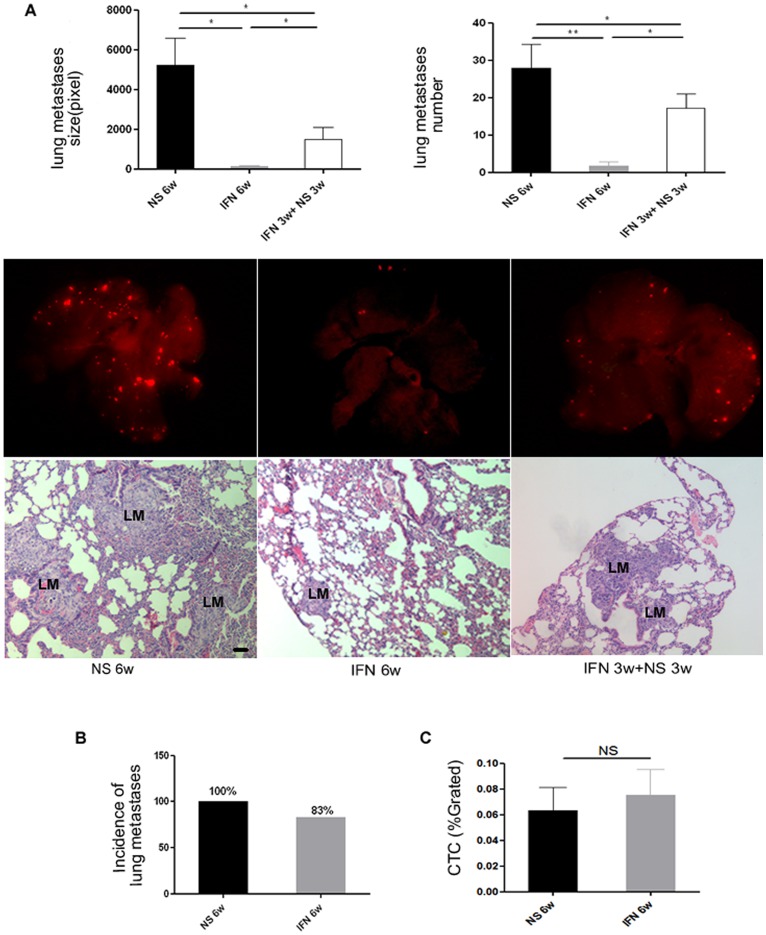
IFN-α inhibited lung metastasis number and size; however, it did not reduce CTCs. Recovery of lung metastasis was induced by IFN-α withdrawal. (**A**) Six-week administration of IFN-α inhibited the number and size of lung metastases, and after IFN-α withdrawal, lung metastasis resumed in terms of both number and size (bars, SEM; **P<0.01, *P<0.05). (Middle) Representative lung tissue from the NS group and IFN-α treatment and withdrawal groups. The metastatic foci are shown in red (upper, HCCLM3 cells with RFP) and H&E staining (lower), black bars, 50 µm. (**B**) Six-week administration of IFN-α did not reduce the incidence of lung metastasis (83% versus 100%, for IFN-α and NS groups, respectively), and (**C**) CTCs (0.075%±0.020% versus 0.063%±0.018%, *P* = 0.574, for IFN-α and control groups, respectively). LM, lung metastasis.

As shown in Figure 1A, the number and size of the lung metastatic lesions of the IFN-α–treated mice were smaller than those of the controls (lung metastasis number: 1.75±1.0 versus 28.0±6.3, *P* = 0.008; metastasis size [pixels]: 116.8±72.2 versus 5226.4±1355.7, *P* = 0.020). However, no significant difference was found in the incidence of lung metastasis between the two groups (83% [5/6] versus 100% [6/6], *P* = 1.000; Fig. 1B). These findings implied that most mice still had lung metastasis regardless of IFN-α treatment, but the severity of lung metastasis was greatly decreased by IFN-α treatment.

### IFN-α Treatment Did Not Reduce the Number of CTCs

CTC arrest in the lung is one of the important steps in lung metastasis, which can be achieved with high efficiency [25]. No significant difference in the number of CTCs (labeled by RFP and detected by flow cytometry) was found between IFN-α–treated and control groups (0.075%±0.020% versus 0.063%±0.018%, *P* = 0.574, Fig. 1C).

### IFN-α Treatment Did Not Reduce Expression of Angiogenic and Proliferation-Related Factors in the Lung Metastatic Foci

To determine whether angiogenesis and cell proliferation in the metastatic tumor cells in the lungs were affected by IFN-α, we used real-time PCR with human-specific primers to detect the expression of several angiogenic and proliferation-related factors that are prominently reduced in primary tumors, as reported in our previous study[14], including VEGF-A, PDGF-A, IL-6, and PCNA. We found a higher expression of angiogenic factors and PCNA in lung metastatic foci in the IFN-α–treated group compared with the untreated controls (2.88±0.30 versus 0.02±0.01, *P* = 0.011 for VEGF-A; 3.40±0.22 versus 0.54±0.19, *P* = 0.000 for PDGF-A; 0.08±0.02 versus 0.02±0.01, *P* = 0.014 for IL-6; 2.54±0.25 versus 2.61± 0.33, *P* = 0.784 for PCNA, expressed in 2^−ΔCT^, respectively).

### IFN-α Inhibited Macrophage Infiltration and MMP-9 Expression in the Lung Tissues

**Figure 2:**
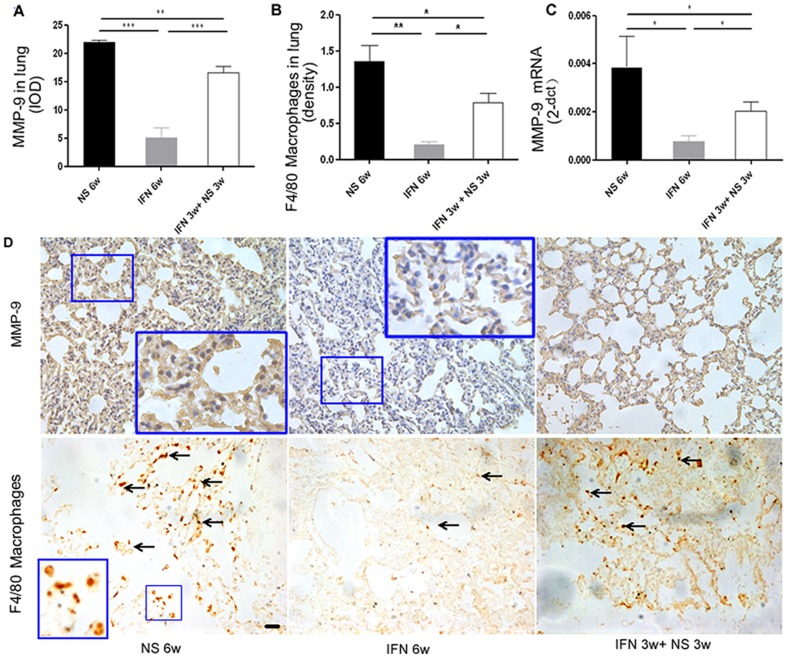
Inhibition of macrophage infiltration and MMP-9 expression in the lung tissue induced by IFN-α; recovery of macrophages and MMP-9 in the lung after IFN-α withdrawal. (**A–C**) Quantification of MMP-9 in lung tissue of the IFN-α group, NS group, and IFN-α withdrawal group by immunohistochemistry staining (**A**) and real-time PCR (**C**). (**B**) Quantification of macrophages in lung tissue using anti-F4/80 antibody by immunohistochemistry staining (bars, SEM; ***P<0.001, **P<0.01, * *P*<0.05). (**D**) Representative pictures of MMP-9 and macrophage immunohistochemistry staining in the lung; macrophages are indicated by arrows. Black bars, 50 µm.

Because the angiogenic and proliferation-related factors in the lung metastatic foci were not inhibited by IFN-α treatment, we hypothesized that lung microenvironment is an important determinant of the fate of lung metastasis. The expression of MMP-9, one of the key players involved in tumor metastases [33] and ‘premetastatic niche’ formation [26], was examined using immunohistochemistry. The results showed that MMP-9 expression in the lung tissues was much lower in the IFN-α–treated mice compared with the untreated mice (mean IOD: 5.1±1.7 versus 21.9±0.4, *P*<0.000; Table 1; Fig. 2A, D). Real-time PCR using the mouse-specific primer also confirmed the lower MMP-9 RNA level in the lung tissue in the IFN-α–treated mice (5.0-fold lower than the untreated mice, *P* = 0.034; Fig. 2C).

**Table 1:** Macrophage infiltration and MMP-9 expression in lung tissue associated with different treatment modalities.

Group	Treatment	Macrophages (%)	MMP-9 (IOD)
Tumor-bearing	NS 6w	1.36%±0.21%	21.9±0.4
	IFN-α 3w+NS 3w	0.79%±0.13%	16.5±1.2
	IFN-α 6w	0.20%±0.04%	5.1±1.7
Non–tumor-bearing	NS 6w	1.13%±0.04%	20.8±0.3
	IFN-α 3w+NS 3w	0.72%±0.03%	14.1±1.2
	IFN-α 6w	0.12%±0.03%	3.8±1.2
IFN-α pretreatment	NS 9w	1.68%±0.00%	34.9±0.1
	IFN-α 3w+NS 6w	1.10%±0.00%	19.0±0.2

3w, 3 weeks; 6w, 6 weeks.

Macrophage infiltration in the lung tissue may be responsible for MMP-9 expression by means of direct production [27] or cooperation between macrophages and pulmonary endothelial cells [26], [27]. To ascertain whether macrophages in the lung tissue were affected by IFN-α, macrophages were counted after anti-F4/80 antibody staining (Fig. 2B, D). The results showed that the number of macrophages in IFN-α–treated mice was significantly lower compared with that in the untreated mice (macrophages density: 0.20%±0.04% versus 1.36%±0.21%, *P* = 0.0058, Table 1). The number of macrophages and expression of MMP-9 in the lungs were also correlated (*cc* = 0.648, *P* = 0.000 and *cc* = 0.504, *P* = 0.000 for IFN-α- and NS-treated groups, respectively).

To ascertain whether IFN-α administration affected the endogenous IFN-α expression in lung tissue, we detected endogenous IFN-α expression using the mouse-specific primers and found comparable small quantities of endogenous IFN-α in lung tissue from both groups (0.06±0.01 versus 0.04±0.01, *P* = 0.613, expressed in 2^−ΔCT^, respectively).

### Lung Metastasis, Macrophage Infiltration, and MMP-9 Expression in the Lung after IFN-α Withdrawal

We found that withdrawal of IFN-α resulted in an increased number and size of lung metastases compared with continuous IFN-α treatment for 6 weeks (number: 17.2±3.8 versus 1.75±1.0, *P* = 0.011; size [pixels]: 1483.2±598.1 versus 116.8±72.2; *P* = 0.014; Fig. 1A). However, the number of CTCs was comparable between the continuous treatment group and the withdrawal group (0.050%±0.010% versus 0.075%±0.020%, *P* = 0.237).

We found that MMP-9 expression and macrophage infiltration in the lung tissue in the IFN-α withdrawal group were higher compared with those in the continuous IFN-α group (immunohistochemistry staining, MMP-9 expression, 16.5±1.2, *P* = 0.0007, Table 1; Fig. 2A, D; macrophage, 0.79%±0.13%, *P* = 0.013, Table 1; Fig. 2B, D). The number of macrophages and the intensity of MMP-9 expression were correlated (*cc* = 0.601, *P* = 0.000 and *cc* = 0.552, *P* = 0.000 for continuous IFN-α and withdrawal group, respectively). Real-time PCR using the mouse-specific primer also detected an increased MMP-9 RNA level derived from lung tissue in the IFN-α withdrawal group compared with the continuous IFN-α group (2.40-fold higher, *P* = 0.038; Fig. 2C). Moreover, tumor angiogenesis indicated by mRNA expression of VEGF-A, PDGF-A, and IL-6 in the lung detected by real-time PCR using the human-specific primers was still much less in the IFN-α withdrawal group than in the continuous group (0.03±0.04 versus 2.88±0.30, *P* = 0.025; 0.04±0.02 versus 3.40±0.22, *P* = 0.004; 0.02±0.02 versus 0.08±0.02, *P* = 0.0007 for VEGF-A, PDGF-A, and IL-6 in IFN-α withdrawal and continuous groups, expressed in 2^−ΔCT^, respectively). Moreover, mRNA expression of PCNA was similar in both groups (2.64±0.32 versus 2.54±0.25, *P* = 0.823, expressed in 2^−ΔCT^, respectively).

### Inhibitory Effect of IFN-α on Macrophages and MMP-9 Expression in Lung Tissue Was Independent of Primary Tumor

**Figure 3:**
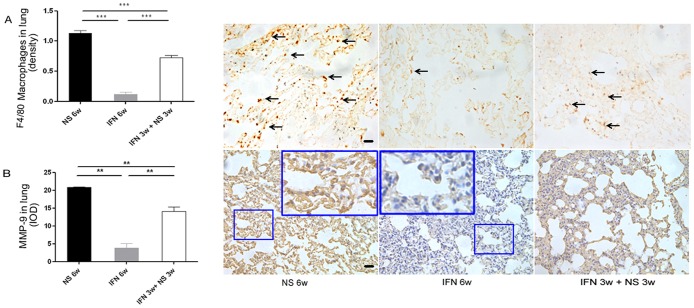
Inhibition on macrophages and MMP-9 in the lung by IFN-α was independent of primary tumor. (**A and B**) Quantification of macrophages and MMP-9 in lung tissue from mice without tumor by immunohistochemistry staining. Both were directly reduced by IFN-α, and the reversal of both was observed after IFN-α withdrawal; reductions were still much less than the control group. (**Right**) Representative lung tissue from three groups. In upper panels macrophages are indicated by arrows; lower panels show MMP-9 expression; bars, SEM; **P<0.01, ***P<0.001. Black bars, 50 µm.

To ascertain whether IFN-α had a direct impact on MMP-9 expression and macrophage infiltration, mice without tumors were treated with IFN-α. Compared with NS-treated mice, both macrophages (0.12%±0.03% versus 1.13%±0.04%, *P* = 0.0001, Table 1; Fig. 3A) and MMP-9 (3.8±1.2 versus 20.8±0.3, *P* = 0.0038; Table 1; Fig. 3B) were significantly reduced in IFN-α–treated mice. Furthermore, the number of macrophages and intensity of MMP-9 expression were also correlated (*cc* = 0.617, *P* = 0.000 and *cc* = 0.547, *P* = 0.000 for IFN-α and NS groups, respectively). The reversal of macrophage infiltration (0.72%±0.03%, *P* = 0.013) and MMP-9 (14.1±1.2, *P* = 0.0007) in the lung was observed in the withdrawal group (Table 1; immunohistochemistry staining, Fig. 3A, B), as compared with the continuous 6-week IFN-α treatment, and the number of macrophages and the intensity of MMP-9 expression were also correlated (*cc* = 0.663, *P* = 0.000 and *cc* = 0.604, *P* = 0.000 for continuous IFN-α and withdrawal groups, respectively). Therefore, macrophage infiltration and MMP-9 expression in the lung tissue were directly inhibited by IFN-α, irrespective of the presence of tumor.

**Figure 4:**
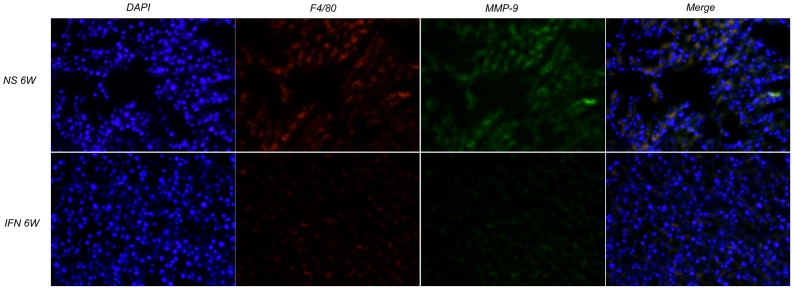
IFN-α reduced MMP-9 positive macrophages. The F4/80 (the marker of macrophages, red) and MMP-9 (green) signals were due to TR- and FITC-labeled antibodies, respectively, using single-layer projections in a confocal microscope. Nuclei were labeled by DAPI (blue); ×20 objective magnification.The immunofluorescence assays showed macrophages may be one of the major sources of MMP-9 in lung tissues, and IFN-α reduced MMP-9 positive macrophages.

Furthermore, we found most macrophages were positive for MMP-9 by double immunofluorescence methods, suggesting macrophages may be one of the major sources of MMP-9 in lung tissues. Furthermore, we also found IFN-α reduced MMP-9 positive macrophages (Fig. 4).

### Pretreatment with IFN-α Inhibited Experimental Lung Metastasis

**Figure 5:**
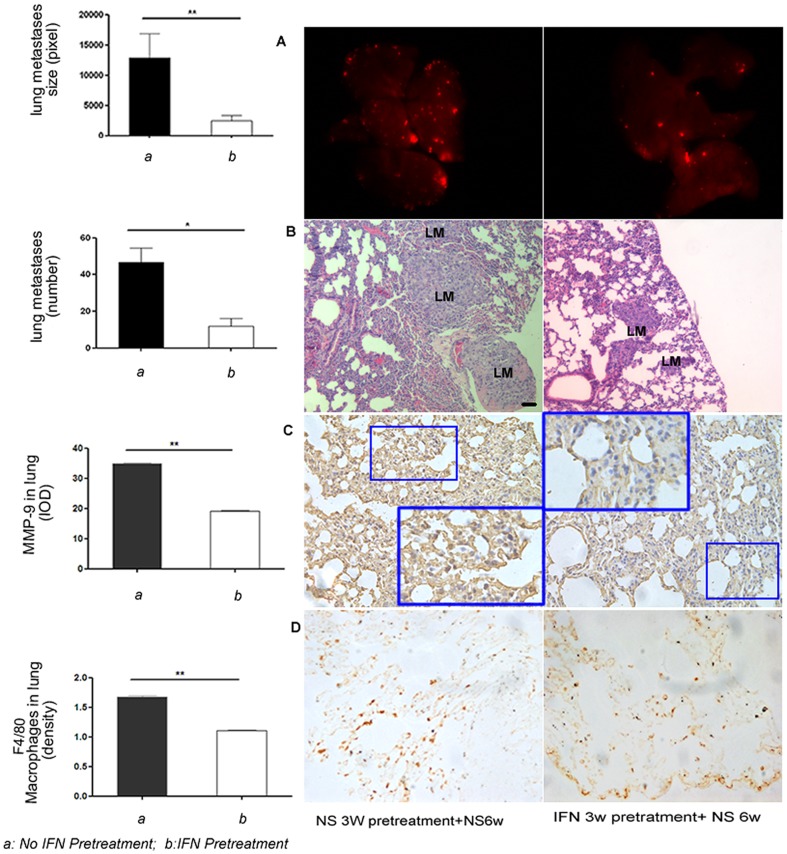
Pretreatment with IFN-α inhibited experimental lung metastasis. After 3 weeks of pretreatment with IFN-α and NS, mice received a tail-vein injection of 1×10^6^ RFP-LM3 cells. Thereafter, both groups received NS for another 6 weeks. The number and size of metastatic foci were remarkably smaller in IFN-α–pretreated mice compared with the NS-pretreated mice. Representative lung from the two groups; the metastatic foci show in red (**A**, HCCLM3 cells with RFP) and H&E staining (**B**). Immunohistochemistry study showed that MMP-9 (**C**) and macrophage infiltration (**D**) in the IFN-α–pretreated group were less than in the NS-pretreated group. Black bars, 50 µm. LM, lung metastasis.

After pretreatment with IFN-α for 3 weeks, mice received a tail vein injection of RFP-LM3 cells (1.0×10^6^). We found the incidence of lung metastasis in IFN-α–pretreated mice was similar compared with the NS-pretreated mice (4/5 versus 5/5); however, the number and size of metastatic foci were remarkably smaller in IFN-α–pretreated mice compared with the NS-pretreated mice (number: 11.8±4.2 versus 46.8±15.3, *P* = 0.021; size [pixels]: 2489.8±838.1 versus 12,803.3±4016.1, *P* = 0.007 for IFN-α- and NS-pretreated groups, respectively; Fig. 5).

Immunohistochemistry study showed that MMP-9 (19.0±0.2 versus 34.9±0.1, *P* = 0.001, Table 1) and macrophage infiltration (1.10%±0.00% versus 1.68%±0.00%, *P* = 0.001, Table 1) in the IFN-α–pretreated group were less than in the NS-pretreated group, and the number of macrophages and intensity of MMP-9 expression were also correlated (*cc* = 0.711, *P* = 0.000 and *cc* = 0.587, *P* = 0.000 for the IFN-α and NS pretreatment groups, respectively). Meanwhile, the mouse origin MMP-9 RNA level in the lungs of the IFN-α–pretreated group was 1.9-fold lower than in the NS-pretreated group (*P* = 0.032).

### Administration of IFN-α associated a shift from M2 to M1 polarization in lung environment

**Figure 6:**
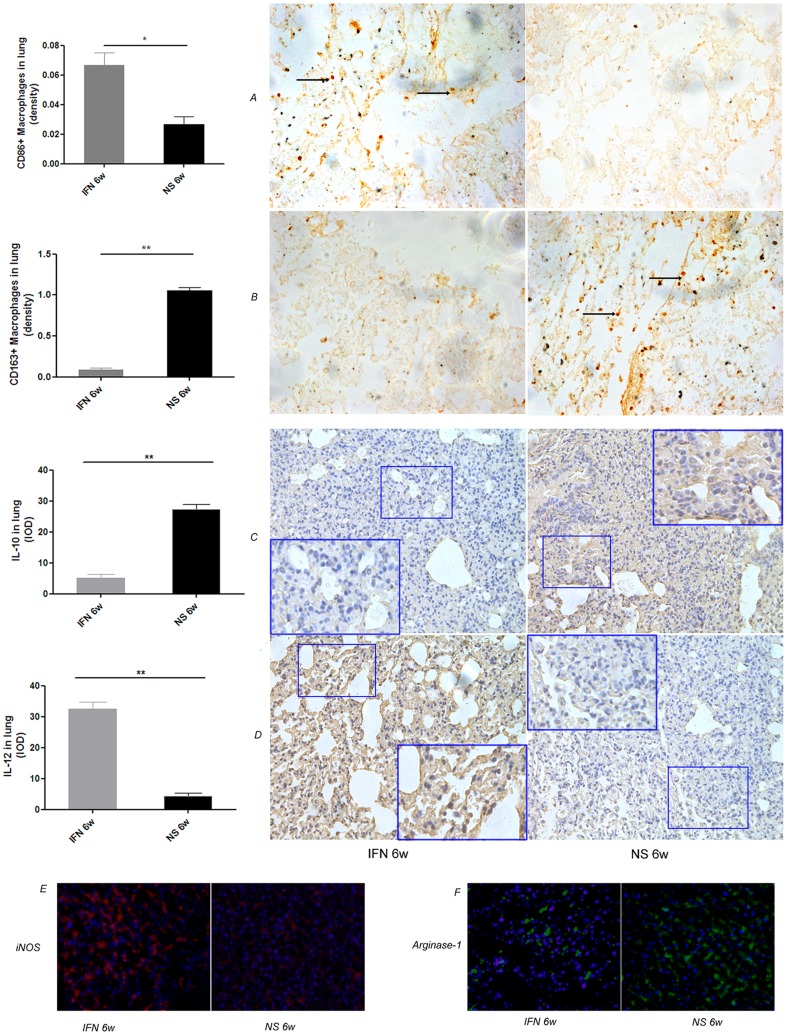
Administration of IFN-α was associated with a shift from M2 to M1 polarization in the lung environment. In the experiment without tumor burden, after 6 weeks of IFN-α treatment, immunohistochemistry staining indicated IFN-α–treated lung tissue had much higher expression of CD86 (**A**) and IL-12 (**D**) and much lower expression of CD163(**B**) and IL-10 (**C**), immunofluorescence assays assessing M1(**E**, iNOS, red) and M2 (**F**, Arg-1, green) macrophage polarity, which indicated IFN-α treatment caused a shift in the macrophage phenotype (shift from M2 to M1) within the lung environment. Bars, SEM; **P<0.01;*P<0.05.

To determine whether IFN-α treatment elicited a shift of macrophage phenotype from M2 to M1 within the lung environment, mice without tumors were treated with IFN-α and NS for 6 weeks; we detected the expression of CD86, iNOS and IL-12 (the markers of M1 macrophages) and CD163, Arg-1 and IL-10 (the markers of M2 macrophages) and in the lung tissues. Figure 6 showed that most macrophages from IFN-α–treated lung tissue had an M1 phenotype, in terms of much higher expression of both CD86 (0.067± 0.008 versus 0.027± 0.005, *P* = 0.023, Fig. 6A) and iNOS (red in IF staining; RT-PCR, 3.34±0.02 versus 0.23±0.06, *P* = 0.002, expressed in 2^−ΔCT^) and much lower expression of both CD163(0.10±0.02 versus 1.05±0.01, *P* = 0.009, Fig. 6B) and Arg-1 (green in IF staining; RT-PCR, 0.43±0.03 versus 3.47±0.02, *P* = 0.015, expressed in 2^−ΔCT^); furthermore, the shift in macrophages was also elicited within the lung environment, that nearly all IFN-α–treated lung tissues had higher expression of IL-12 and lower expression of IL-10 (IL-10 expression, 5.2±1.2 versus 27.2±1.6, *P* = 0.005, Fig. 6C; IL-12 expression, 32.5±2.2 versus 4.3±1.0, *P* = 0.003; Fig. 6D). These results were supported by the findings from RT-PCR detection of IL-10 and IL-12 of mouse origin (0.05±0.02 versus 4.20±0.12, *P* = 0.012; 2.14±0.06 versus 0.38±0.30, *P* = 0.025; for IL-10 and IL-12 in IFN-α and NS group, expressed in 2^−ΔCT^, respectively), which supported the possibility that IFN-α treatment induces a shift from M2 to M1 polarization in the lung tissues.

## Discussion

In the present study, we found that the growth of HCC metastatic foci in the lung was suppressed because of a direct modulation of the lung microenvironment by IFN-α treatment, probably through inhibition of both MMP-9 expression and macrophage infiltration in the lung tissues.

The cascade of metastasis includes early steps, such as cells from a primary tumor entering into the circulation, survival of the cells in the circulation, and arrest in a new organ, followed by later steps, such as extravasation into the target tissue, initiation and maintenance of growth, and angiogenesis of the metastatic tumor. In vivo videomicroscopy and cell-fate analysis have shown that the early steps in metastasis are efficient, while the later steps are inefficient because the ability to grow is dictated by molecular interactions of the cells with the environment in the secondary organ [25]. This pattern is consistent with our findings that the incidence of lung metastasis and the number of CTCs in IFN-α–treated and untreated groups were similar, whereas the growth of lung metastasis was inhibited by IFN-α treatment.

However, we found that several prominent angiogenic factors such as VEGF-A, PDGF-A, and IL-6 in the metastatic tumor cells were not inhibited, while all these factors were significantly inhibited by IFN-α in the primary tumor site[14]. This suggested that IFN-α selected for a subpopulation of tumor cells that were distinct from the predominant population of primary tumor cells and may have been resistant to IFN-α at the secondary metastatic organs. Therefore, it seems unlikely that inhibition of growth of lung metastasis was mediated by the anti-angiogenic property of IFN-α.

The most interesting finding is that IFN-α treatment could directly modulate the lung microenvironment to inhibit the growth of lung metastasis; the macrophages and MMP-9 expression in the lungs could be the target of IFN-α treatment. There are two types of macrophages, presenting either a tumoricidal effect (M1) or a pro-tumor effect (M2) [34]. In our present study, based on the cytokine expression pattern in lung tissue, we found IFN-α treatment was associated with a shift of macrophage phenotype from M2 to M1 within the tumor environment. This suggests that IFN-α treatment may promote a macrophage-related tumoricidal effect on metastatic cells in lung. However, this result also needs to be confirmed by studying the expression of other cytokines located on the macrophages.

The pro-tumor effect of M2 type macrophages is associated with their production of MMPs, urokinase-type plasminogen activator (uPA), uPA receptor (uPAR), and others, of which MMP-9 has been proved to have complex effects, including induction of the angiogenic switch and release of growth factors [35], [36]. MMP-9 could also be expressed by other host cells in lung environment such as other inflammatory cells (neutrophils) [27] and lung endothelial cells [26]. Although tumor cells could be another source of MMP-9, we proved MMP-9 of mouse origin was down-regulated by IFN-α treatment. In addition, double-staining immunofluorescence methods supported macrophages being the major source of MMP-9 in the lungs. Furthermore, the intensity of MMP-9 expression and number of infiltrating macrophages were statistically correlated, which supported macrophages being a major source of MMP-9 in the lung.

The role of MMP-9 in lung metastasis is conflicting in different studies. Some reports considered macrophage-derived MMP-9 in the lung tissue to be a critical component for priming of the premetastatic niche, such that primary tumor–stimulated lung macrophages could efficiently cooperate with endothelial cells to up-regulate MMP-9 via a VEGF receptor-1–dependent pathway to facilitate survival and growth of metastatic lesions in the lung [26], [28]. Other studies showed that MMP-9, produced by both macrophages and neutrophils (majority), was pro-angiogenic for lung metastatic lesion [27]. Our present study showed that IFN-α preconditioned lung, associated with reduced MMP-9 expression, is not a promising site for the incoming tumor cells. However, other molecules in the lung environment that may be associated with IFN-α treatment should also be studied, such as MMP-12, MMP-13, RhoGDI2, and metadherin [37], [38], [39].

The present study implied that withdrawal of IFN-α treatment may result in increased MMP-9 expression and macrophage infiltration in the lung and consequently more lung metastasis or tumor recurrence, which is consistent with our clinical studies [24]. Like many other anti-angiogenesis drugs, the duration of treatment is an unsolved question.

In conclusion, the present study demonstrated that IFN-α treatment could significantly suppress lung metastasis; however, we also found that CTCs arrested in the lung had a largely limited fate due to the ‘hostile’ lung microenvironment induced by IFN-α treatment. This suggests great clinical potential for IFN-α in preventing or retarding lung metastasis. This treatment may be suitable for patients with inoperable lung metastasis associated with HCC, and it may reduce postoperative extrahepatic lung metastasis.

## Supporting Information

Table S1 **Primers used for real time-PCR.**
(DOC)
