# Supplementary material for: Direct Transformation of Lung Microenvironment by Interferon-α Treatment Counteracts Growth of Lung Metastasis of Hepatocellular Carcinoma
Source: PLoS One. 2013 Mar 18;8(3):e58913. doi: 10.1371/journal.pone.0058913 (PMC3601095; doi:10.1371/journal.pone.0058913)
Supplement: Table S1 — Primers used for real time-PCR. (DOC) [file pone.0058913.s001.doc]

***Supplementary Table 1. Primers used for real time-PCR***

| Gene name | Orientation | Primer sequence (5′–3′) | Species |
| --- | --- | --- | --- |
| *IL-6* | Forward | TCTCCACAAGCGCCTTCG | Human |
| *IL-6* | Reverse | CTCAGGGCTGAGATGCCG | Human |
| *PDGF-A* | Forward | ACACGAGCAGTGTCAAGTGC | Human |
| *PDGF-A* | Reverse | GGCTCATCCTCACCTCACAT | Human |
| *VEGF-A* | Forward | GCAGAAGGAGGAGGGCAGAATC | Human |
| *VEGF-A* | Reverse | GGCACACAGGATGGCTTGAAG | Human |
| *PCNA* | Forward | CCATCCTCA AGA AGGTGTTGG | Human |
| *PCNA* | Reverse | GTGTCCCATATCCGCAATTTTAT | Human |
| *GAPDH* | Forward | CCATCACCATCTTCCAGG | Human |
| *GAPDH* | Reverse | ATGAGTCCTTCCACGATAC | Human |
| *MMP-9* | Forward | CCTACTGCGGGCTCTTCTGA | Mouse |
| *MMP-9* | Reverse | CCCTGTAATGGGCTTCCTCT | Mouse |
| *IL-10* | Forward | GCTCTTACTGACTGGCATGAG | Mouse |
| *IL-10* | Reverse | CGCAGCTCTAGGAGCATGTG | Mouse |
| *IL-12* | Forward | ATGTGTCAATCACGCTACCTC | Mouse |
| *IL-12* | Reverse | TCAGGCGGAGCTCAGATAG | Mouse |
| *IFN-α* | Forward | TGCAACCCTCCTAGACTCATTCT | Mouse |
| *IFN-α* | Reverse | CCAGCAGGGCGTCTTCCT | Mouse |
| *iNOS* | Forward | CGAAACGCTTCACTTCCAA | Mouse |
| *iNOS* | Reverse | TGAGCCTATATTGCTGTGGCT | Mouse |
| *Arg-1* | Forward | AACACGGCAGTGGCTTTAACC | Mouse |
| *Arg-1* | Reverse | GGTTTTCATGTGGCGCATTC | Mouse |
| *GAPDH* | Forward | GCACAGTCAAGGCCGAGAAT | Mouse |
| *GAPDH* | Reverse | GCCTTCTCCATGGTGGTGAA | Mouse |
